# Gut microbiome and major depressive disorder: insights from two-sample Mendelian randomization

**DOI:** 10.1186/s12888-024-05942-6

**Published:** 2024-07-08

**Authors:** Qian Zhao, Ancha Baranova, Hongbao Cao, Fuquan Zhang

**Affiliations:** 1 Department of Psychiatry, The Affiliated Brain Hospital of Nanjing Medical University, Nanjing, 210029 China; 2 School of Systems Biology, George Mason University, Fairfax, 22030 USA; 3 Research Centre for Medical Genetics, Moscow, 115478 Russia; 4 Institute of Neuropsychiatry, The Affiliated Brain Hospital of Nanjing Medical University, Nanjing, 210029 China

**Keywords:** Mendelian randomization, GWAS, Gut microbiome, Major depressive disorder, Brain-gut axis

## Abstract

**Background:**

Existing evidence suggests that alterations in the gut microbiome are closely associated with major depressive disorder (MDD). We aimed to reveal the causal relationships between MDD and various microbial taxa in the gut.

**Methods:**

We used the two-sample Mendelian randomization (TSMR) to explore the bidirectional causal effects between gut microbiota and MDD. The genome-wide association studies summary results of gut microbiota were obtained from two large consortia, the MibioGen consortium and the Dutch Microbiome Project, which we analyzed separately.

**Results:**

Our TSMR analysis identified 10 gut bacterial taxa that were protective against MDD, including phylum *Actinobacteria*, order *Clostridiales*, and family *Bifidobacteriaceae* (OR: 0.96 ∼ 0.98). Ten taxa were associated with an increased risk of MDD, including phyla *Firmicutes* and *Proteobacteria*, class *Actinobacteria*, and genus *Alistipes* (OR: 1.01 ∼ 1.09). On the other hand, MDD may decrease the abundance of 12 taxa, including phyla *Actinobacteria* and *Firmicutes*, families *Bifidobacteriaceae* and *Defluviitaleaceae* (OR: 0.63 ∼ 0.88). MDD may increase the abundance of 8 taxa, including phylum *Bacteroidetes*, genera *Parabacteroides*, and *Bacteroides* (OR: 1.12 ∼ 1.43).

**Conclusions:**

Our study supports that there are mutual causal relationships between certain gut microbiota and the development of MDD suggesting that gut microbiota may be targeted in the treatment of MDD.

**Supplementary Information:**

The online version contains supplementary material available at 10.1186/s12888-024-05942-6.

## Background

The human gut is a complex ecosystem consisting of bacteria, viruses, fungi, and other microorganisms collectively known as the gut microbiota. The human gut contains a few thousand bacterial species, which are usually described using the taxonomic units of phylum, order, family, genus, species, and strain. Most studied representatives of the gut microbiota of healthy adults include phyla *Firmicutes*, *Bacteroidetes*, *Actinobacteria*, and *Proteobacteria*, with *Firmicutes* and *Bacteroidetes* appearing to be jointly dominant (up to 90%) [1]. Gut microbes are multifunctional, dynamic community, that participates in a range of physiological processes critical to host health, making important contributions to energy homeostasis, metabolism, intestinal epithelial health, immune activity, and neurodevelopment. Detrimental changes in the diversity and relative abundance of microbial taxa and species that make up the gut flora have been termed “gut dysbiosis” and have been linked to a variety of diseases, including inflammatory bowel disease, asthma, obesity, dementia, and autism [2, 3]. The human gut microbiota is influenced by environmental and other factors, and it is noteworthy that the importance of the host genetic component in shaping the composition of an individual’s microbiome has also been demonstrated [4].

Worldwide, depression is a severely disabling public health problem associated with significant distress, morbidity, mortality, and costs. The lifetime prevalence of major depressive disorder (MDD) is 16.2% [5]. The World Health Organization predicts that by 2030, MDD will be the leading cause of disease burden worldwide [6]. Only 30–40% of patients are relieved by treatment with a single antidepressant medication, leaving nearly 60–70% of patients without an optimal response [7]. Currently, MDD is recognized as a multifactorial disorder with a definite role in multiple etiological factors such as genetic predisposition, stress, and inflammation [8]. MDD is commonly comorbid, and may even increase the risks for the development of other diseases, or facilitate their progression [9–11]. Studies have shown that MDD is heritable to a moderate degree. The heritability ranges from 31 to 42% and is thought to rely on a complex interaction of multiple risk genes [12]. In some cases, genetic factors can promote or even trigger depression.

In recent years, a growing body of research has revealed that the gut microbiota and the brain communicate in a bidirectional way, influencing each other, and these studies have also demonstrated the existence of the gut-brain axis [13, 14]. Observational studies have shown differences in the composition of the gut microbiota between healthy individuals and patients with MDD compared to healthy controls [15]. However, these differences did not reach uniformity across these studies. Observational studies focusing on the diversity of the gut microbiota are unable to make causal inferences about which specific bacterial taxa are responsible for population differences [16]. Mendelian randomization (MR) is the use of genetic variation as an instrumental variable (IV) to detect and quantify causality in observational epidemiological studies. It can avoid some of the problems of traditional observational studies by minimizing confounders, including age, drug or environmental exposure, and reverse causation [17]. This analytical approach is now widely used to infer causality from a genetic perspective [18–21]. In this study, we used a two-sample MR (TSMR) analysis to explore the causal effect between gut microbiota and MDD [22].

## Methods

### Genome-wide association studies (GWAS) summary datasets

The GWAS summary results used for this analysis were all from publicly available data. The summary data on the gut microbiota were obtained from two sources: the international consortium MibioGen (MibioGen) and the Dutch Microbiome Project (Dutch). MibioGen [23] is a GWAS summary statistic involving 18,340 participants: a total of 212 taxa belonging to 35 families, 20 orders, 16 classes, 9 phyla, and 131 genera. Of these, 15 unknown families and genera of gut microbial taxa were excluded. The MibioGen dataset is a large multi-ethnic GWAS collaborative project consisting of 18,340 participants from 16 cohorts from various countries, including the United States, Canada, Israel, South Korea, Germany, Denmark, the Netherlands, Belgium, Sweden, Finland, and the United Kingdom. The summary data for Dutch [24] are mainly from the Dutch Microbiome Project: this project studied the composition and function of the gut microbiome in 8208 individuals. We only used GWAS data for 207 taxa and did not use the relevant metabolic pathway sections. GWAS data for MDD [25] (*N* = 807,553, Ncase = 246,363, Ncontrol = 561,190) were analyzed using data from three of the largest existing genetic studies of depression: the UK Biobank study (UK Biobank), 23andMe, and the Psychiatric Genomics Consortium. Ethical approval was obtained in all original studies.

### TSMR analysis

In R (version 4.0.5), we performed the TSMR between the gut microbiome and MDD. The analysis employed three complementary methods integrated into TwoSampleMR (version 0.5.6) [22], including inverse variance weighted (IVW), weighted median, and MR-Egger. These methods have distinct assumptions regarding horizontal pleiotropy. The IVW model served as our primary TSMR approach [26], assuming zero intercepts and yielding consistent causality estimates through fixed-effects meta-analysis. The MR-Egger model assumes that pleiotropic effects are independent and applies weighted linear regression of outcome coefficients to exposure coefficients. Horizontal pleiotropy was assessed using MR-Egger-based P_pleiotropy (P_pleiotropy > 0.05) [26]. However, when MR-Egger suggests pleiotropy, we used the MRPRESSO analysis as a complementary method to the pleiotropy test. When the Raw-based Causal Estimate is in the same direction as the beta effect value of IVW and the Global Test_P > 0.05, it means that the results are robust and have no horizontal pleiotropy [27]. The heterogeneities were gauged by both I^2^ statistics and Cochran’s Q test (both I^2^ > 0.25 and *P* < 0.05) [28]. Finally, we performed a leave-one-out (LOO) sensitivity analysis and excluded IVs one by one to test whether our MR results were robust. An IVW-based *P* < 0.05 determined a significant correlation between the gut microbiome and MDD.

In TSMR analysis of the causal effects of MDD on the gut microbiota, single-nucleotide polymorphisms (SNPs) with genome-wide significance (*P* < 5 × 10^− 8^) were selected as IVs and further pruned using a clumping r^2^ cutoff of 0.001 within a 10 Mb window, using the 1000 Genomes Project Phase 3 (EUR). In reverse causal effect analysis, a relatively relaxed threshold of 1 × 10^− 5^ was used to select IVs because there were fewer IVs. We assessed the genetic instrument strength by using F statistics [29]. When performing TSMR analysis, we deleted the SNPs that did not exist in the outcome dataset and palindromic SNPs with intermediate allele frequencies. We reconcile each pair of exposure and outcome datasets by aligning the effect alleles of exposure and outcome.

## Results

### TSMR analysis

**Fig. 1:**
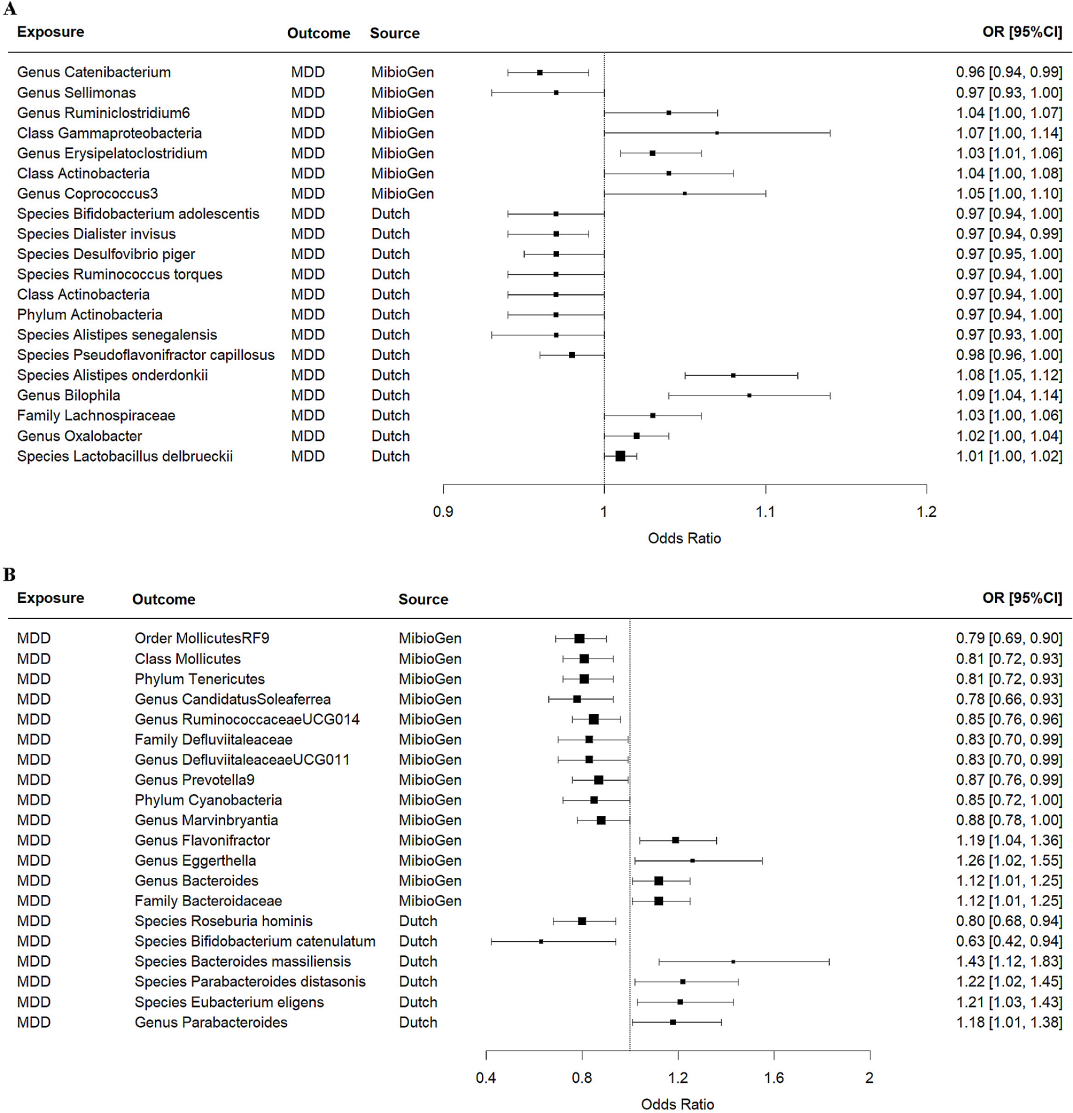
Causal effects between the gut microbiome and MDD (forest plot). **(A)** Causal effects of the gut microbiome on MDD. **(B)** Causal effects of MDD on the gut microbiome. CI: confidence interval; MDD: major depressive disorder; OR: odds ratio; *P*: *P* value

**Fig. 2:**
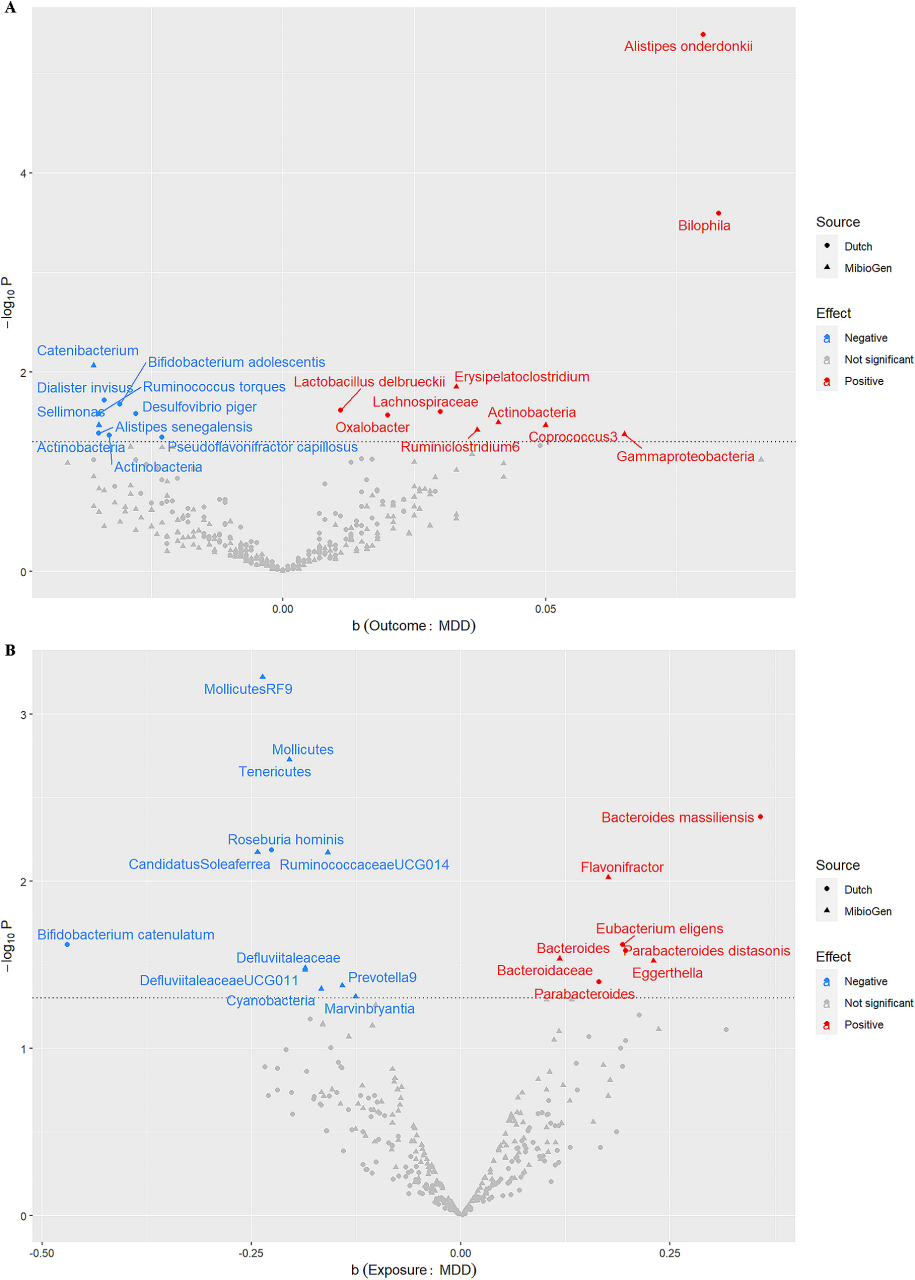
Causal effects between the gut microbiome and MDD (scatter plot). **(A)** Causal effects of the gut microbiome on MDD. **(B)** Causal effects of MDD on the gut microbiome. b: MR estimate; MDD: major depressive disorder; *P*: *P* value

Our TSMR results revealed a causal effect between gut microbiota and MDD, and there were differences in the results of two different gut microbiota datasets. (Tables 1 and 2; Fig. 1, and Fig. 2).

**Table 1:** TSMR analyses reveal causal effects of the gut microbiome on MDD.

Exposure	Outcome	Source	OR [95%CI]	*P*
Genus *Catenibacterium*	MDD	MibioGen	0.96 [0.94–0.99]	8.55E-03
Genus *Sellimonas*	MDD	MibioGen	0.97 [0.93-1.00]	0.034
Genus *Ruminiclostridium6*	MDD	MibioGen	1.04 [1.00-1.07]	0.038
Class *Gammaproteobacteria*	MDD	MibioGen	1.07 [1.00-1.14]	0.042
Genus *Erysipelatoclostridium*	MDD	MibioGen	1.03 [1.01–1.06]	0.014
Class *Actinobacteria*	MDD	MibioGen	1.04 [1.00-1.08]	0.032
Genus *Coprococcus3*	MDD	MibioGen	1.05 [1.00-1.10]	0.034
Species *Alistipes.onderdonkii*	MDD	Dutch	1.08 [1.05–1.12]	4.07E-06
Genus *Bilophila*	MDD	Dutch	1.09 [1.04–1.14]	2.56E-04
Species *Bifidobacterium adolescentis*	MDD	Dutch	0.97 [0.94-1.00]	0.021
Species *Dialister invisus*	MDD	Dutch	0.97 [0.94–0.99]	0.019
Species *Desulfovibrio piger*	MDD	Dutch	0.97 [0.95-1.00]	0.026
Species *Ruminococcus torques*	MDD	Dutch	0.97 [0.94-1.00]	0.026
Class *Actinobacteria*	MDD	Dutch	0.97 [0.94-1.00]	0.043
Phylum *Actinobacteria*	MDD	Dutch	0.97 [0.94-1.00]	0.043
Species *Alistipes senegalensis*	MDD	Dutch	0.97 [0.93-1.00]	0.041
Species *Pseudoflavonifractor capillosus*	MDD	Dutch	0.98 [0.96-1.00]	0.045
Family *Lachnospiraceae*	MDD	Dutch	1.03 [1.00-1.06]	0.025
Genus *Oxalobacter*	MDD	Dutch	1.02 [1.00-1.04]	0.027
Species *Lactobacillus delbrueckii*	MDD	Dutch	1.01 [1.00-1.02]	0.024

CI: confidence interval; MDD: major depressive disorder; OR: odds ratio; P: P value

**Table 2:** TSMR analyses reveal causal effects of MDD on the gut microbiome

Exposure	Outcome	Source	OR [95%CI]	*P*
MDD	Order *MollicutesRF9*	MibioGen	0.79 [0.69–0.90]	6.01E-04
MDD	Class *Mollicutes*	MibioGen	0.81 [0.72–0.93]	1.87E-03
MDD	Phylum *Tenericutes*	MibioGen	0.81 [0.72–0.93]	1.87E-03
MDD	Genus *CandidatusSoleaferrea*	MibioGen	0.78 [0.66–0.93]	6.69E-03
MDD	Genus *RuminococcaceaeUCG014*	MibioGen	0.85 [0.76–0.96]	6.73E-03
MDD	Family *Defluviitaleaceae*	MibioGen	0.83 [0.70–0.99]	0.033
MDD	Genus *DefluviitaleaceaeUCG011*	MibioGen	0.83 [0.70–0.99]	0.034
MDD	Genus *Prevotella9*	MibioGen	0.87 [0.76–0.99]	0.042
MDD	Phylum *Cyanobacteria*	MibioGen	0.85 [0.72-1.00]	0.044
MDD	Genus *Marvinbryantia*	MibioGen	0.88 [0.78-1.00]	0.049
MDD	Genus *Flavonifractor*	MibioGen	1.19 [1.04–1.36]	9.48E-03
MDD	Genus *Eggerthella*	MibioGen	1.26 [1.02–1.55]	0.030
MDD	Genus *Bacteroides*	MibioGen	1.12 [1.01–1.25]	0.029
MDD	Family *Bacteroidaceae*	MibioGen	1.12 [1.01–1.25]	0.029
MDD	Species *Roseburia hominis*	Dutch	0.80 [0.68–0.94]	6.50E-03
MDD	Species *Bifidobacterium catenulatum*	Dutch	0.63 [0.42–0.94]	0.024
MDD	Species *Bacteroides massiliensis*	Dutch	1.43 [1.12–1.83]	4.10E-03
MDD	Species *Parabacteroides distasonis*	Dutch	1.22 [1.02–1.45]	0.026
MDD	Species *Eubacterium eligens*	Dutch	1.21 [1.03–1.43]	0.024
MDD	Genus *Parabacteroides*	Dutch	1.18 [1.01–1.38]	0.040

CI: confidence interval; MDD: major depressive disorder; OR: odds ratio; P: P value

TSMR results from MibioGen suggest that genera *Catenibacterium* and *Sellimonas* reduce MDD risk (OR: 0.96 ∼ 0.97, *P* ≤ 0.034), but classes *Actinobacteria* and *Gammaproteobacteria*, genera *Erysipelatoclostridium*, *Ruminiclostridium6*, and *Coprococcus3* increase MDD risk (OR: 1.03 ∼ 1.07, *P* ≤ 0.042). Dutch data suggest that phylum *Actinobacteria*, class *Actinobacteria*, species *Bifidobacterium adolescentis*, *Dialister invisus*, *Desulfovibrio piger*, *Ruminococcus torques*, *Alistipes senegalensis*, and *Pseudoflavonifractor capillosus* (OR: 0.97 ∼ 0.98, *P* ≤ 0.045) were associated with a reduced risk of MDD, but family *Lachnospiraceae*, genera *Oxalobacter* and *Bilophila*, species *Lactobacillus delbrueckii*, and *Alistipes onderdonkii* (OR: 1.01 ∼ 1.09, *P* ≤ 0.027) were associated with an increased risk of MDD (Table 1; Fig. 1A, and Fig. 2A). Notably, the causal effect of class *Actinobacteria* on MDD is reversed in MibioGen (OR = 1.04, 95%CI: 1.00-1.08, *P* = 0.032) and Dutch (OR = 0.97, 95%CI: 0.94-1.00, *P* = 0.043).

Reverse causal results from MibioGen suggest that the genetic liability to MDD is associated with a reduction in phyla *Cyanobacteria* and *Tenericutes*, class *Mollicutes*, order *MollicutesRF9*, family *Defluviitaleaceae*, genera *CandidatusSoleaferrea*, *RuminococcaceaeUCG014*, *DefluviitaleaceaeUCG011*, *Prevotella9*, and *Marvinbryantia* (OR: 0.79 ∼ 0.88, *P* ≤ 0.049), as well as an increase in family *Bacteroidaceae*, genera *Flavonifractor*, *Eggerthella*, and *Bacteroides* (OR: 1.12 ∼ 1.26, *P* ≤ 0.030). MDD may decrease species *Roseburia hominis*, and *Bifidobacterium catenulatum* (OR: 0.63 ∼ 0.80, *P* ≤ 0.024), as well as increase genus *Parabacteroides*, species *Bacteroides massiliensis*, *Parabacteroides distasonis*, and *Eubacterium eligens* (OR: 1.18 ∼ 1.43, *P* ≤ 0.040) in Dutch (Table 2; Fig. 1B, and Fig. 2B).

MR sensitivity analysis showed that the directions of causal effect estimates across the set of applied techniques were largely the same. No horizontal pleiotropy was detected in the result of the MR-Egger model and MRPRESSO analysis (Supplementary Tables 1–3). The Cochran’Q test and the I^2^ statistics showed no heterogeneity between most of the effect estimates, with one exception of genus *Sellimonas* (Supplementary Tables 1 and Supplementary Table 2). Each IV has an F statistic > 10, indicating no weak instruments (Supplementary Table 4). The robustness of some results was confirmed by the LOO sensitivity analyses, including those for phylum *Tenericutes*, class *Mollicutes*, order *MollicutesRF9*, genera *CandidatusSoleaferrea*, *RuminococcaceaeUCG014*, *Flavonifractor*, and *Bilophila*, and species *Alistipes onderdonkii*, *Bacteroides massiliensis*, *Roseburia hominis*, *Bifidobacterium catenulatum*, *Eubacterium eligens*, and *Parabacteroides distasonis*. For other datasets, the LOO analysis suggests that single or multiple SNPs with potential to influence the causal effect; therefore, these results should be interpreted with caution (Supplementary Fig. 1 and Supplementary Fig. 2).

## Discussion

Our study shows that some gut microbiota is associated with a reduced risk of MDD and also identifies flora that can increase the risk of MDD and that MDD can also alter the composition of the gut microbiota, most of which is localized to taxa such as phyla *Actinobacteria*, *Bacteroidetes*, and *Firmicutes*, classes *Actinobacteria*, *Bacteroidia*, and *Clostridia*, orders *Bacteroidales* and *Clostridiales*, families *Bacteroidaceae*, *Bifidobacteriaceae*, and *Lachnospiraceae*, genera *Alistipes* and *Bifidobacterium*.

Many studies echoing our results have shown that remodeling of the gut microbiota caused by genetic variation and MDD can act as functional modulators of each other. A preclinical study suggests that stress-induced depressive-like behavior in mice can be attenuated by fecal microbiome transplantation by a mechanism partially attributed to the gut microbiota [30]. An MR study demonstrated a causal effect of increased *Morganella* on MDD. This was thereafter validated observationally with follow-up records up to 16 years, yielding consistent results on the effect [31]. In another MR study, the investigators found that class *Actinobacteria*, its family *Bifidobacteriaceae*, and its genus *Bifidobacterium* had a protective causal effect on MDD, while genus *Ruminococcus1* may be antiprotective against MDD pathogenesis [32]. In this TSMR study, their results on the causal effect of class *Actinobacteria* on MDD were the opposite of ours. Their gut microbiota data also came from MibioGen, but their sample size of the GWAS data for MDD was only 480,359, while our sample size was more than 1.5 times that. Our analysis showed that class *Actinobacteria* had the opposite effect on the risk of MDD in two different gut microbiota data. This may be because MibioGen is a multi-ethnic large-scale GWAS that coordinated 24 cohorts from the United States, Canada, Israel, South Korea, Germany, Denmark, the Netherlands, Belgium, Sweden, Finland, and the United Kingdom, while Dutch analyzed data from volunteers from the northern Netherlands. In addition, the MibioGen (2021) data are slightly outdated compared to Dutch (2022). The role of class *Actinobacteria* in MDD needs more research.

A cross-sectional study found significant gut microbiota disturbances in patients with depression, with a significant reduction in *Firmicutes* [33]. In another systematic review and meta-analysis of observational studies, it was shown that several taxa at the family and genus levels, specifically, family *Prevotellaceae*, genus *Corprococcus*, and *Faecalibacterium*, were decreased in MDD when compared to non-depressed controls [16]. Recently, a retrospective cohort study emphasized that levels of several *Enterobacteriaceae* differed significantly between MDD patients and healthy controls [34]. In addition, there are MR studies supporting that MDD alters the composition of the gut microbiota [35].

We learned some possible explanations for the relevant mechanisms behind the causal links revealed by our research. Gut bacteria influence processes such as neuroinflammation, stress axis activation, neurotransmission, and neurogenesis through their multiple functions [36]. Studies conducted in humans and animal models suggest that both immune dysregulation and inflammation play a crucial role in the pathophysiology of MDD [37]. Increasing evidence suggests that a dysregulated gut microbiota may secrete large amounts of lipopolysaccharides and amyloid proteins, which may lead to increased intestinal permeability or increased blood-brain barrier permeability during aging [38]. Gut inflammation may lead to systemic changes in inflammation, which reaches the central nervous system in different ways to modulate inflammatory pathways, especially inducing activation of microglia, which can induce depression [39, 40]. Gut bacteria can synthesize important neurotransmitters, which can alter the expression of several central nervous system receptors by modulating serotonin, thus enabling them to directly influence brain excitability and function and exert epigenetic control over gene expression [41]. Gut bacteria can produce metabolites such as short-chain fatty acids (SCFAs) that may have neuroactive properties. It has been shown to reduce depressive-like behavior in mice by inhibiting microglia activation and neuroinflammation. It has been demonstrated that the reason why MDD patients with relatively high abundance of some gut flora (e.g., *Blautia*, *Coprococcus*, and *Bifidobacterium*), which are associated with the production of SCFAs, responded to selective serotonin reuptake inhibitors (SSRIs) antidepressants may be that SCFA maintains high levels of 5-hydroxytryptamine synthesized precursors by upregulating the expression of Tryptophan hydroxylases 1 in vitro, thereby enhancing the antidepressant-like effects of SSRIs antidepressants [42, 43].

This study also suggests that patients with MDD undergo significant changes in the gut microbiota after treatment with SSRIs antidepressants. Whether the changes in gut microbiota composition that MDD can cause as shown in our study involve mediation by antidepressants is not known at this time. A 2019 study reported that *Lachnospiraceae* were more abundant in SSRIs-treated mice when compared to the control group [44]. Another review highlighted that there were no significant high levels of *Lactobacillus* after controlling for medications [45]. Consumption of high-fat and animal protein diets was also associated with elevated abundance of *Actinobacteria* [46]. Low carbohydrate intake with a lack of disaccharide metabolism was once hypothesized to be involved in the reduction of *Prevotellaceae* in patients with autism [47].

Most studies did not control for diet and psychotropic drugs. Drug therapy and diet remain important sources of inter-study differences in the composition of gut microorganisms between MDD and controls. In future studies, these factors should be considered. Increasing evidence supports the efficacy of various microbiota-targeting therapies in alleviating depression, including dietary interventions, gut microbiota transplantation, probiotics, etc. [48]. Our study once again demonstrates that aimed at gut microbiota remains a feasible avenue for modification of depression phenotypes. Future attempts to use gut microbiota profiles for MDD prevention, diagnosis, and treatment will require more research to unravel and further explain the mechanisms behind these effects.

Due to the use of MR analysis, we were able to avoid confounding factors and reverse causality to a greater extent than is possible in the frame of observational research. We explored in a hypothesis-free manner to ensure diversity of results. We used GWAS data from two large gut microbiomes with small overlaps and sizable sample sizes to increase statistical power. Multiple sensitivity analyses ensured the robustness of our results. At the same time, we recognize some limitations of our study. MR analyses may be biased by multiple effects, so we tested the MR hypotheses using various models. We did not make multiple-test corrections to adjust each p-value, which could increase the likelihood of false positives. We analyzed only genetic factors for both diseases and therefore caution should be exercised in interpreting the results. The gut microbiota may be influenced by environmental factors such as dietary habits or acquired health conditions, which are mostly of low heritability. Knowing that we still could not test whether genetic tools were associated with these confounding factors. In addition, the use of cross-ancestry data makes it impossible to generalize when interpreting results and applying them to other ethnic groups.

## Conclusion

Our study suggests that certain gut microbiota contribute to the risk of MDD, while MDD may affect the composition of the gut microbiota.

### Electronic supplementary material

Below is the link to the electronic supplementary material.

Supplementary Material 1

Supplementary Material 2

## Data Availability

All data generated or analyzed during this study are included in this published article and its supplementary information files.

## Abbreviations

MDD: Major depressive disorder
MR: Mendelian randomization
TSMR: Two-sample MR
GWAS: Genome-wide association studies
IVW: Inverse variance weighted
IVs: Instrumental variables
SNPs: Single-nucleotide polymorphisms
SCFAs: Short-chain fatty acids
SSRIs: Selective serotonin reuptake inhibitor
