# Supplementary material for: Gut microbiome and major depressive disorder: insights from two-sample Mendelian randomization
Source: BMC Psychiatry. 2024 Jul 8;24:493. doi: 10.1186/s12888-024-05942-6 (PMC11232322; doi:10.1186/s12888-024-05942-6)

**Supplementary Figure 1. Leave-one-out sensitivity analysis for** **gut microbiome on MDD.** A. Genus *Oxalobacter* (Dutch). B. Genus *Bilophila* (Dutch). C. Phylum *Actinobacteria* (Dutch). D. Species *Bifidobacterium adolescentis* (Dutch). E. Species *Alistipes onderdonkii* (Dutch). F. Species *Alistipes senegalensis* (Dutch). G. Species *Lactobacillus delbrueckii* (Dutch). H. Species *Pseudoflavonifractor capillosus* (Dutch). I. Species *Dialister invisus* (Dutch). J. Species *Desulfovibrio piger* (Dutch). K. Species *Ruminococcus torques* (Dutch). L. Class *Actinobacteria* (Dutch). M. Family *Lachnospiraceae* (Dutch). N. Class *Actinobacteria* (MibioGen). O. Class *Gammaproteobacteria* (MibioGen). P. Genus *Catenibacterium* (MibioGen). Q. Genus *Coprococcus3* (MibioGen). R. Genus *Erysipelatoclostridium* (MibioGen). S. Genus *Ruminiclostridium6* (MibioGen). T. Genus *Sellimonas* (MibioGen).


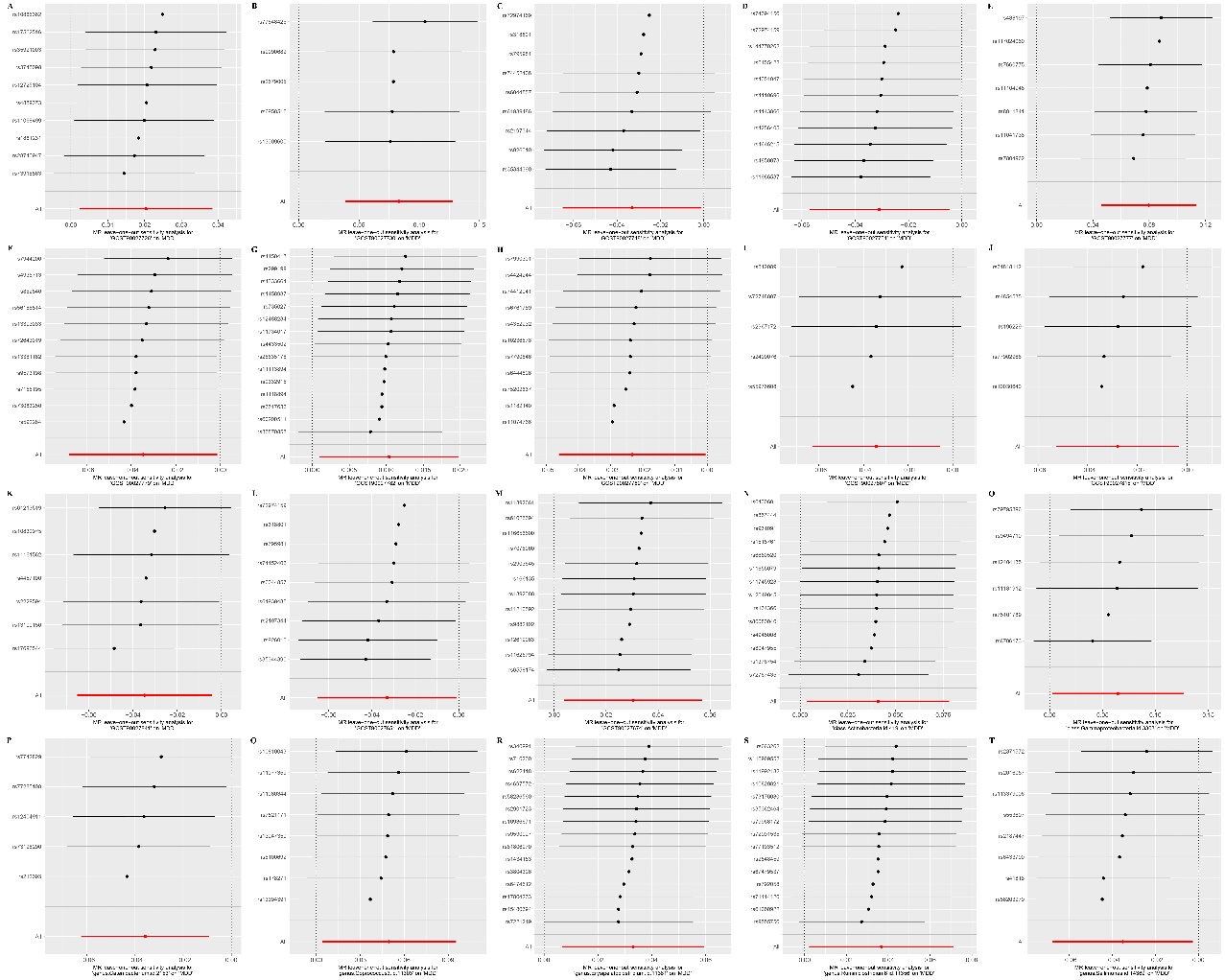


**Supplementary Figure 2. Leave-one-out sensitivity analysis for** **MDD on gut microbiome.** A. Species *Eubacterium eligens* (Dutch). B. Species *Bacteroides massiliensis* (Dutch). C. Species *Roseburia hominis* (Dutch). D. Genus *Parabacteroides* (Dutch). E. Species *Bifidobacterium catenulatum* (Dutch). F. Species *Parabacteroides distasonis* (Dutch). G. Phylum *Cyanobacteria* (MibioGen). H. Phylum *Tenericutes* (MibioGen). I. Family *Bacteroidaceae* (MibioGen). J. Family *Defluviitaleaceae* (MibioGen). K. Genus *Bacteroides* (MibioGen). L. Genus *CandidatusSoleaferrea* (MibioGen). M. Genus *DefluviitaleaceaeUCG011* (MibioGen). N. Genus *Eggerthella* (MibioGen). O. Genus *Flavonifractor* (MibioGen). P. Genus *Marvinbryantia* (MibioGen). Q. Genus *Prevotella9* (MibioGen). R. Genus *RuminococcaceaeUCG014* (MibioGen). S. Order *MollicutesRF9* (MibioGen). T. Class *Mollicutes* (MibioGen).


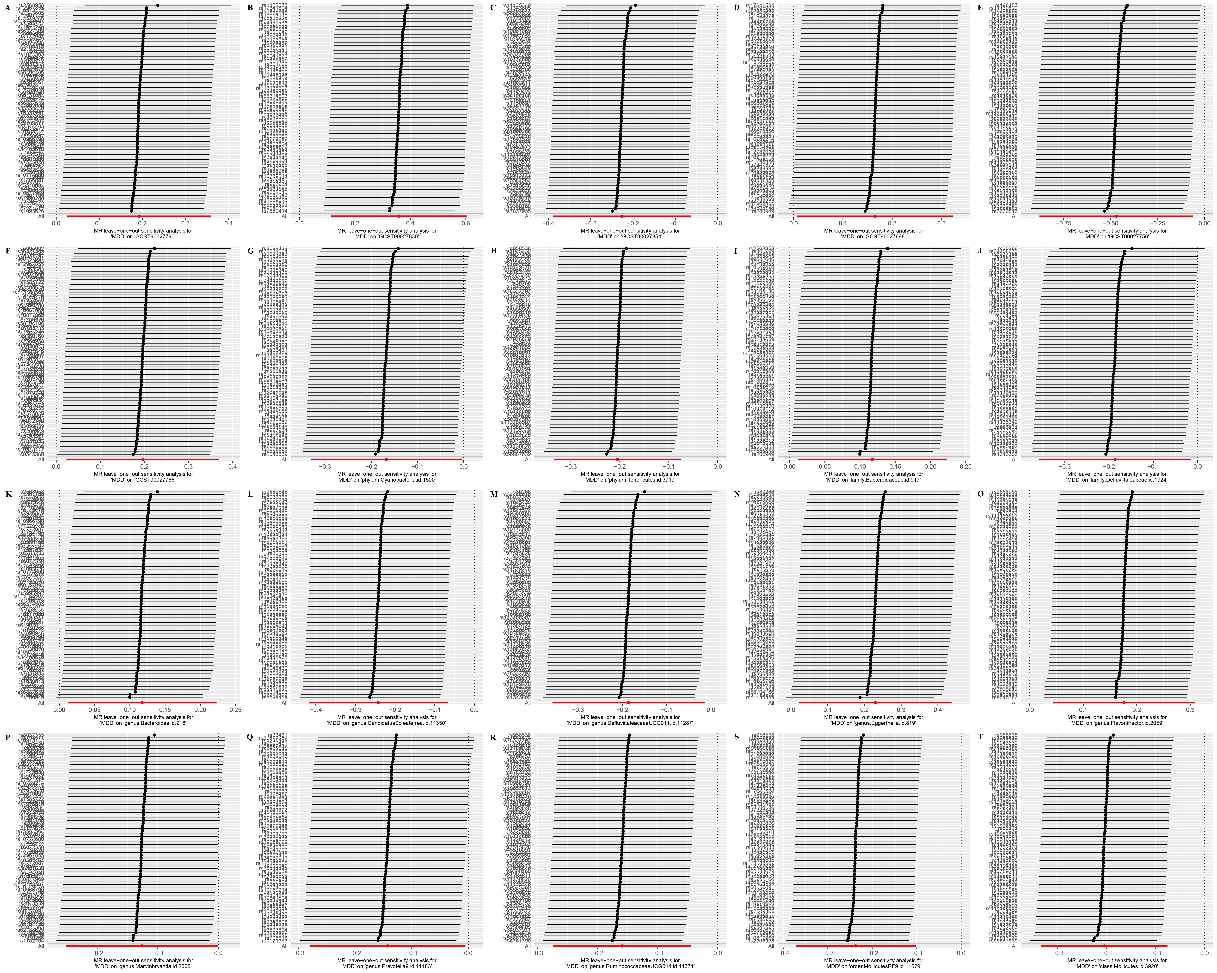

Supplement: Supplementary file 1 — Supplementary Material 1 [file 12888_2024_5942_MOESM1_ESM.docx]
